# Reward-based prioritization in working memory is distinct from recency and due to a resource trade-off

**DOI:** 10.3758/s13423-025-02810-6

**Published:** 2025-12-09

**Authors:** Timothy J. Ricker, Christopher J. Cagna, Tien T. Tong, Ekaterina Dobryakova, Joshua Sandry

**Affiliations:** 1 Department of Psychology, University of South Dakota, Vermillion, SD USA; 2 Center for Traumatic Brain Injury Research, Kessler Foundation, East Hanover, NJ USA; 3 Perelman School of Medicine, The University of Pennsylvania, 3400 Civic Center Boulevard, Philadelphia, PA 19104 USA; 4 Psychology Department, Montclair State University, 1 Normal Ave, Montclair, NJ 07043 USA

**Keywords:** Working memory, Focus of attention, Recency effect, Reward, Prioritization, Drift diffusion model

## Abstract

Prioritized items within working memory often show higher accuracy or faster response times at test. This prioritization benefit is thought to reflect the memory representation being within the focus of attention. The literature on prioritization effects lacks consistency in whether prioritization benefits manifest as a benefit to speed, accuracy, or both, implying that some findings may reflect a speed-accuracy tradeoff rather than improved memory representation. In the present work, we use drift-diffusion modeling to test two theoretical questions regarding prioritization effects without the possibility of a confounding speed-accuracy tradeoff. First, we test whether prioritization effects rely on shifting limited working memory resources away from non-prioritized items. Second, we test whether prioritization and recency effects are two distinct mechanisms or two ways to enter the focus of attention. We find that prioritization effects do reflect shifts in limited working memory resources and that prioritization and recency are two distinct mechanisms. These findings are augmented by model fits suggesting differential effects of prioritization at the perceptual/motor and cognitive levels.

**Supplementary Information:**

The online version contains supplementary material available at 10.3758/s13423-025-02810-6.

## Introduction

Humans have the ability to flexibly prioritize their focus of attention (FOA) to high-value non-recent information within working memory (WM), the system that maintains temporarily active information (Cowan et al., 2023). This effect is robust across verbal and visual stimuli (Allen & Ueno, 2018; Atkinson et al., 2021; Atkinson et al., 2018; Atkinson et al., 2022; Hitch et al., 2018; Hu et al., 2016; Hu et al., 2014; Sandry & Ricker, 2020; Sandry et al., 2014; Sandry et al., 2020; Zheng et al., 2022) and can also benefit long-term memory depending on context (Atkinson et al., 2023; Jeanneret et al., 2023; Sandry et al., 2020). Prioritization is induced experimentally using different reward schemes, whereby some to-be-remembered information is assigned a higher value than other information. It is debated whether reward-based prioritization reflects different cognitive mechanisms from retro-cueing (Atkinson et al., 2018; Jeanneret et al., 2023; Zhang & Lewis-Peacock, 2023). Here, we explore the mechanisms related to reward-based prioritization effects.

Within a sequentially presented list, early-list stimuli are responded to faster or more accurately when they are prioritized. The prioritization effect is not due to distinctiveness of encoding (Sandry & Ricker, 2020). Interestingly, when the last item is prioritized, it seems to receive a joint benefit of both prioritization and recency (i.e., better memory for the most recently presented stimulus) compared to the last item (recency) under no prioritization (Sandry & Ricker, 2020; Sandry et al., 2014; Sandry et al., 2020). The recency benefit to memory performance is typically thought to reflect a benefit from being within the FOA (McElree, 2006; McElree & Dosher, 1989; Nee & Jonides, 2008, 2011; Öztekin et al., 2010), making prioritization and recency two paths to the same end in many theoretical models. Many popular models of WM treat the FOA as binary (items are either in it or out of it), implying that a recent item should not benefit much, if at all, from prioritization (Adam et al., 2017; Barrouillet & Camos, 2015; Cowan, 1995; Hardman et al., 2017; Oberauer & Bialkova, 2009; Rouder et al., 2008; Zhang & Luck, 2008). The additional benefit of prioritizing the final item in a list instead suggests that prioritization and recency effects may depend on different cognitive mechanisms that each improve memory strength. The exact nature of the prioritization mechanism in contrast to recency remains unresolved.

Previous investigations into the prioritization effect have shown evidence for an attentional resource tradeoff across to-be-remembered stimuli (Atkinson et al., 2018; Atkinson et al., 2023; Brissenden et al., 2023; Hu et al., 2014; Sandry & Ricker, 2020; Sandry et al., 2014; Sandry et al., 2020). That is, increased attention allocated to a prioritized stimulus results in a performance benefit for the prioritized stimulus and a corresponding performance detriment for the other stimuli in the list. Whether the effects manifest as changes in reaction time (RT) or accuracy is inconsistent and the potential for speed-accuracy tradeoffs introduces interpretative issues (Ratcliff & McKoon, 2008). Prioritization may also speed perceptual or motor/response processing, further obscuring the contributions of speed-accuracy tradeoffs in attention and memory-related processing. Interpreting prioritization effects and attributing a resource tradeoff explanation is difficult using these traditional dependent measures when speed and accuracy are not held constant or considered together. Fortunately, a well-developed computational modelling approach, the drift-diffusion model (DDM)^1^ (Ratcliff, 1978; Ratcliff & McKoon, 2008; Ratcliff et al., 2016), provides more nuanced information that circumvents these limitations.

The DDM assumes that information is accumulated over time (i.e., drift rate) and a response is initiated after accumulating enough information to cross a decision boundary or threshold. In the context of a memory recognition task, better memory performance is evident with larger drift rates representing faster and more reliable accumulation of evidence. DDM also provides boundary separation parameters to quantify information about speed-accuracy tradeoffs, and non-decision times, or the residual information representing the strength of stimulus encoding, motor response execution, and other processing unrelated to the decision itself (jointly estimated with a single parameter). With respect to the current investigation, drift rates provide a unique measure of cognitive ability that indexes the quality of the memory representation available at the time of testing. The drift rate can be used to test the degree that prioritization incurs a resource tradeoff across list positions and whether prioritization and recency are overlapping or distinct processes that are uncontaminated by a speed-accuracy tradeoff or changes in perceptual or motor processing.

One previous experiment (Grogan et al., 2022, Experiment 4) examined reward-based prioritization effects with a Linear Ballistic Accumulator model, a modeling framework similar to DDM that also produces estimates of drift rates and decision thresholds. Grogan et al. (2022) used a two-item visual WM task and varied whether each item was prioritized across trials. When prioritization cues were given before the memory items, the behavioral data indicated a prioritization-induced speed-accuracy tradeoff with high-reward items being slower and more accurate. When prioritization cues occurred after item presentation, the effect on accuracy was smaller and without a change in RT. Statistical analysis of the Linear Ballistic Accumulator model fit was not central to the paper and insufficient for adjudicating the presence of a prioritization-induced speed-accuracy trade-off or resource trade-off across high- and low-priority items. Given the incomplete and conflicting results, the effects of prioritization in this data set are unclear. The present work addresses these questions in more detail.

## Present experiment

In the present investigation, participants completed a two-alternative forced-choice (2AFC) visual WM task where reward was used to systematically prioritize stimulus positions. On some trials, one list position was presented in red font and associated with a higher point value to induce prioritization within the FOA. Past work has demonstrated that providing points for accurate performance in WM tasks leads to improved performance for the prioritized items (Allen & Ueno, 2018; Atkinson et al., 2018; Jeanneret et al., 2025; Sandry & Ricker, 2020; Sandry et al., 2014; Sandry et al., 2020). The effect of points is likely due to general intrinsic motivational factors increasing interest in performing well (Wang et al., 2024).

To characterize the cognitive processes underlying prioritization, we employed DDM to contrast a Resource Tradeoff model against an Attentional Boost model. The Resource Tradeoff model predicts that the prioritized item will receive a performance benefit, at a cost to the non-prioritized items (i.e., increased attention towards the prioritized stimulus takes away from limited resources allocated to other list positions). The Attentional Boost model predicts that successful prioritization of a stimulus in WM is the result of increased attention, but at no cost to the other items. Within each theoretical model, we further tested whether prioritization and recency depend on shared or separate mechanisms. If prioritization and recency are redundant mechanisms to enter the FOA, we would expect no difference in drift rate between prioritized recent stimuli and non-prioritized recent stimuli, as the result is the same regardless of the path into the FOA. If prioritization and recency are distinct processes, we would expect an additional effect of prioritization, whereby larger drift rates would be evident for prioritized recent stimuli compared to prioritized non-recent stimuli or non-prioritized recent stimuli. Determining which of these models best describes WM performance is key to understanding how reward-based prioritization influences limited WM resources.

## Method

Data for the present experiment are available via the Open Science Framework (OSF) at https://osf.io/sc5g3/. This study received Institutional Review Board (IRB) approval from Montclair State University (MSU). The experiment and analyses were not preregistered.

### Participants

In our past investigations using this paradigm and stimulus combination, we identified reliable within-participant effects with 30 participants and set this as our minimum sample size requirement. Based on our experience with this paradigm, we assumed performance-based exclusions would, on average, reduce the overall sample by about 10.5% (range: 6.9–14.5% exclusion). Additionally, participation required three consecutive lab visits (sessions) to ensure an adequate number of trials to saturate the distribution for our model-based inferences (see *Procedure*). Because we were also interested in evaluating the modulating role of individual differences in mind-wandering and reward sensitivity, we planned to recruit a larger sample than our past work. We posted study timeslots over two academic semesters and included participants with complete data for all three sessions. This resulted in a final sample of 61 participants. All participants were recruited from the MSU Psychology Participant Pool in exchange for partial course credit.

### Materials and design

Participants completed a visual WM probe-recognition experiment with a reward manipulation that directly replicated Experiment 3 of Sandry and Ricker (2020). Visual stimuli were 204 unfamiliar characters that are not easily verbally recoded (Ricker & Cowan, 2014; Sandry & Ricker, 2020). In this study, participants visited the lab and completed three sessions, with each session scheduled exactly 1 week apart. The design was a 3 Session (first, second, third) X 4 Prioritization Position (red stimulus in the First, Middle, or Last list position or no red stimulus in list [Control]) X 3 Probe Position (First, Middle, or Last) within-participant design.

### Procedure

**Fig. 1:**
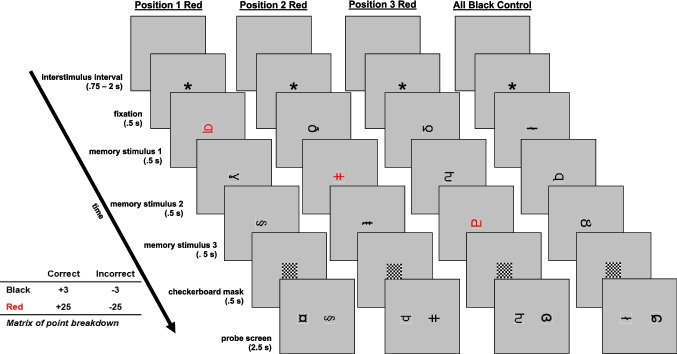
Examples of an experimental trial in each Prioritization Position condition alongside a point breakdown matrix

The procedure is visualized in Fig. 1. The experiment took the form of a visual WM game where participants could earn or lose points for correct or incorrect responses. Each sequentially presented trial began with a blank interstimulus interval (randomized between.75 and 2 s), followed by a fixation asterisk (.5 s) and three to-be-remembered stimuli presented serially at the center of the screen (.5 s each). Next, a checkerboard mask (.5 s) preceded the 2AFC probe recognition/test screen (2.5 s), composed of a matching stimulus (an item from the three-item list) and a non-matching stimulus (an item that was not presented in the three-item list). Match and non-match stimuli appeared on the left and right side of the screen 50% of the time. 2AFC stimuli were always presented in black irrespective of the color of the stimulus during the presentation sequence. After making their selection, participants were presented with a feedback screen (1.5 s) indicating whether they were correct and a running tally of their accumulated points. Stimuli were drawn randomly without replacement from a larger 204-item unfamiliar character stimulus set. Each list position (serial position) was equally likely to be probed at test.

Participants gained points for correct answers and lost points for incorrect answers. On 75% of trials, one item from the three-item list appeared in red and was worth 25 points instead of the standard 3 points for black items. The point breakdown was applied for both gains and losses. Red items were no more likely to be tested than black items. Self-paced breaks occurred every 68 trials. During the break, participants viewed their current accuracy and points along with accuracy and points from previous blocks.

Participants completed ten practice trials with optional opportunities to repeat practice after reviewing instructions and prior to starting the experiment. Experimental trials consisted of a total of 408 experimental trials administered at each session for a total of 1,224 trials per participant (~102 trials per model parameter).

### Post task questionnaires

After each session, participants answered three self-report questions using a 100-point sliding scale anchored between *not at all* and *extremely*: (1) “*How motivated were you to score a high number of points?*”, (2) “*How tired were you while performing the experiment?*”, and (3) “*Overall, how motivated were you throughout the experiment?*” Participants also selected the performance strategy they used from a list of ten possible WM maintenance strategies (Sandry & Ricker, 2020; adapted from Morrison, Rosenbaum, Fair, & Chein, 2016). Including these self-report items served as a direct replication of past research (Sandry & Ricker, 2020) and allowed us to monitor session-related fluctuations across these variables. We chose to include post-task questions to help explain any interactive effects of Session, if present in the behavioral data. A small number of participants with missing self-report responses were omitted in a case-wise fashion independently for each question (adjusted sample size is reflected in degrees of freedom).

Participants also completed two additional individual differences measures, including the reward responsiveness subscale of the Behavioral Inhibition and Activation Scale (BIS/BAS) (Carver & White, 1994) and the Mind-wandering Questionnaire (Mrazek et al., 2013) to capture individual differences in reward sensitivity and mind-wandering, respectively.

### Data preprocessing and exclusionary criteria

Participants were excluded for mean accuracies at/below chance (*N* = 1) or ≤ 2 standard deviations below group-level (*N* = 7) within a session. Participants were also excluded if their mean RT met or exceeded 2 standard deviations shorter/longer than the group-level mean within each session (*N* = 4). This resulted in a final sample of 49 participants included in data analysis. Within each participant, individual trials with RTs shorter than.3 s or longer than 3 standard deviations above the participant’s mean were discarded, resulting in removal of 2.28% of all trials.

### Computational modelling and hierarchical drift-diffusion model (HDDM) parameter estimation

We employed computational modelling with the Bayesian hierarchical drift diffusion model (HDDM) Python toolbox (Wiecki et al., 2013) using the dockerHDDM 1.0.1 implementation (Pan et al., 2024) to evaluate differences across drift-rate and non-decision time as a function of experimental manipulations of Prioritization Position and Probed Position and to evaluate differences in boundary separation across Prioritized Position. To foreshadow, our behavioral data analysis revealed no meaningful interactions by Session, so we did not include Session as a factor in our computational modelling. The HDDM uses Markov Chain Monte Carlo simulation and allows for estimation and recovery of model parameters at the individual subject level that are constrained by the group. For each model, we ran six parallel chains of 4,000 sampling iterations and discarded the first 2,000 iterations of each chain as burn-in. We verified model convergence using Gelman-Rubin statistics (Gelman & Rubin, 1992) and visual examination of trace and convergence plots. We then collapsed the remaining iterations of all six chains into a single posterior distribution of 12,000 iterations. Visual comparison of trace plots and posterior distributions for each individual chain were compared to ensure that there was no multimodality in individual chains or introduced by collapsing across chains into a single posterior distribution.

### Model specification

We created eight models with different parameter specifications. First, we specified four base models by crossing each of our theoretical positions discussed in the **Introduction** (Attentional Boost vs. Resource Tradeoff; Recency/Prioritization mechanisms are equivalent vs. different). We then implemented each of the four models in two structures, one incorporating a speed-accuracy tradeoff and the other without, yielding eight models in total.

**Fig. 2:**
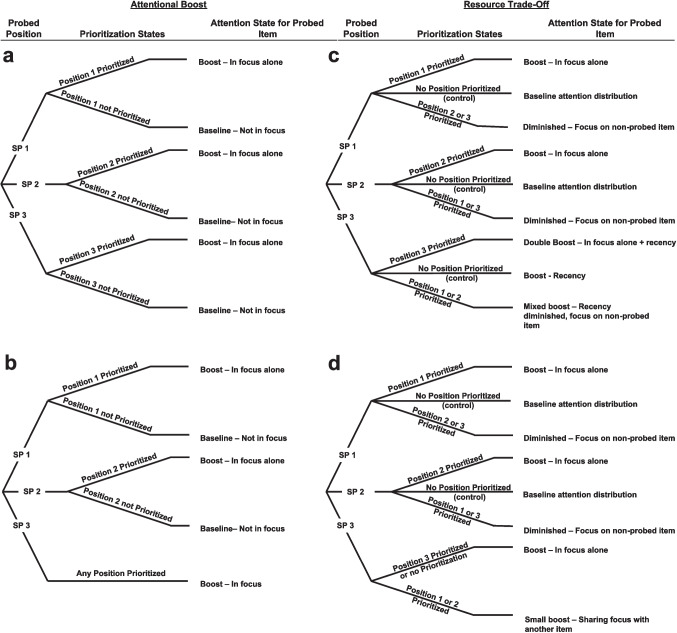
Schematic representations of the four model types demonstrating the different drift-rate and non-decision-time parameters. For each branch ending, there is one drift rate and one non-decision time estimated. The Attentional Boost models are shown on the left where (**a**) prioritization and recency differ and where (**b**) prioritization and recency are equivalent. The Resource Tradeoff models are shown on the right where (**c**) prioritization and recency differ and where (**d**) prioritization and recency are equivalent. See the main text for detailed descriptions. Each serial position (SP) and attention state combination has a unique configuration of drift-rate and non-decision-time parameters

In each model, we allowed drift rate (*v*) and non-decision time (*t*) to vary across conditions as dictated by each of our four theory-based modeling frameworks, detailed in the following paragraphs. The data were error-coded (correct or incorrect), so bias (*z*) was not estimated. The speed-accuracy tradeoff was implemented by allowing boundary separation (*a*) to vary as a function of prioritization condition but not as a function of probe position because participants could not know what item was being probed until after their memory decision had been made. In the no speed-accuracy tradeoff models, a single boundary separation parameter was estimated. The next four paragraphs describe each model’s parameterization in detail. See Fig. 2 for a schematic breakdown of each model’s parameterization. For formal model equations used in parameter estimation, see Wiecki et al. (2013).

*Attention Boost, prioritization and recency differ *(Fig. 2a)*.* In this model, we assume that memory and perceptual/motor characteristics vary depending on which serial position is probed. For each serial position, we assume that items could either be at baseline (during the control condition or when the prioritized position did not match the probed position) or prioritized (when prioritized position matched the probed position). Separate drift-rate and non-decision-time parameters were estimated for each serial position at baseline and when prioritized, resulting in six drift rate and six non-decision-time parameters.

*Attention Boost, prioritization, and recency are equivalent *(Fig. 2b)*.* In this model, we assume that memory and perceptual/motor characteristics vary depending on which serial position is probed. For non-recent serial positions, we assume that items could either be at baseline (during the control condition or when the prioritized position did not match the probed position) or prioritized (when prioritized position matched the probed position). We assume the most recently presented item is in the FOA and so prioritization to bring it into the FOA will have no effect. Separate drift-rate and non-decision-time parameters were estimated for each serial position. For non-recent positions, separate parameters were estimated when each position was at baseline and when each position was prioritized. For the most recent item, only one set of parameters was estimated. This resulted in five drift-rate and five non-decision-time parameters.

*Resource tradeoff, prioritization, and recency differ *(Fig. 2c)*.* In this model, we assume that memory and perceptual/motor characteristics vary depending on which serial position is probed. For each serial position, we assume that items could either be at baseline (when no items were prioritized in the control condition), prioritized (when prioritized position matched the probed position), or diminished (when the prioritized position did not match the probed position). Separate drift-rate and non-decision-time parameters were estimated for each serial position at baseline, when prioritized, and when diminished, resulting in nine drift rate and nine non-decision-time parameters.

*Resource tradeoff, prioritization, and recency are equivalent *(Fig. 2d)*.* In this model, we assume that memory and perceptual/motor characteristics vary depending on which serial position is probed. For non-recent serial positions, we assume that items could be at baseline (when no items were prioritized in the control condition), prioritized (when prioritized position matched the probed position), or diminished (when the prioritized position did not match the probed position). We assume the most recently presented item is always in the FOA at test but could be alone (in the control condition or when the final item was prioritized) or sharing the FOA (when a non-recent item was prioritized), with the latter reducing the FOA benefit. Separate drift-rate and non-decision-time parameters were estimated for each serial position. For non-recent positions, separate parameters were estimated when each position was at baseline, prioritized, and diminished. For the most recent item, separate parameters were estimated when it was alone in the FOA and when it shared the FOA. This resulted in eight drift rate and eight non-decision-time parameters.

### Model selection

Posterior-predictive cumulative-distribution-function quantile plots between parameter estimates and empirical data were visually inspected for each participant to ensure acceptable model fit. To determine the best model, we used Bayesian Predictive Information Criterion (BPIC) to evaluate goodness of model fit (Ando, 2007). BPIC combines the likelihood function with a stringent penalty (larger penalty term than Deviance Information Criterion) for model complexity. Lower BPIC values are indicative of better fit.

## Results

### Behavioral performance

**Fig. 3:**
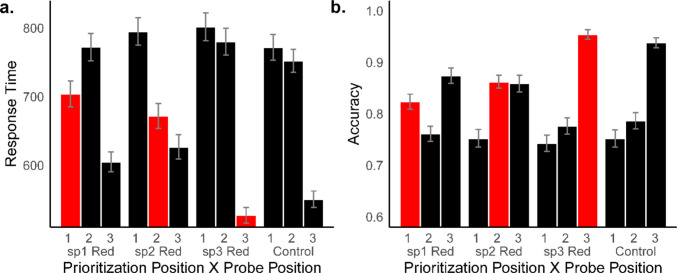
(**a**) Mean response time in milliseconds and (**b**) mean accuracy for probe position and prioritization conditions. Red bars represent prioritized, high point value (25 points) list positions, and black bars represent standard point value (3 points) conditions. Numerical values on x-axis labels represent probed serial positions (sp) with prioritization position label appearing underneath. Error bars represent standard error of the mean

Mean RTs and accuracy are shown in Fig. 3. Bayesian ANOVAs for these behavioral measures are reported in Online Supplement 1.

### Post task questionnaire

Analysis of post task questionnaire data is reported in Online Supplement 2.

### HDDM model comparison

Table 1 provides a breakdown of the BPIC model fit indices quantified for each of the eight models. Inspection of the BPIC values shows a clear win for Model 7, the resource tradeoff model where prioritization and recency represent different mechanisms (Fig. 2c). All attentional boost models demonstrated much larger BPIC, as did all models with a speed-accuracy tradeoff.

**Table 1:** Theoretical model with number of free parameters per participant for drift rate (v), boundary seperation (a), and non-decision time (t) that were allowed to vary for each model. Model fits and model selection were determined using Bayesian Predictive Information Criterion (BPIC). Lower values indicate better model fit

Theoretical Model	HDDM Model	Description	No. of Free Parameters per Participant					BPIC	BPIC Difference from Best Model
			*v*	*a*	*t*	*Total*			
Attentional Boost									
	Model 1	*Prioritization and Recency Differ* *Speed-Accuracy Tradeoff Exists*	6	4	6	(16)		19584	802
	Model 2	*Prioritization and Recency are Equivalent* *Speed-Accuracy Tradeoff Exists*	5	4	5	(14)		20990	2208
	Model 3	*Prioritization and Recency Differ* *No Speed-Accuracy Tradeoff*	6	1	6	(13)		19674	892
	Model 4	*Prioritization and Recency are Equivalent* *No Speed-Accuracy Tradeoff*	5	1	5	(11)		21092	2310
Resource Trade-Off									
	Model 5	*Prioritization and Recency Differ* *Speed-Accuracy Tradeoff Exists*	9	4	9	(22)		19025	243
	Model 6	*Prioritization and Recency are Equivalent* *Speed-Accuracy Tradeoff Exists*	8	4	8	(20)		19188	406
	Model 7	*Prioritization and Recency Differ* *No Speed-Accuracy Tradeoff*	9	1	9	(19)		18782	0
	Model 8	*Prioritization and Recency are Equivalent* *No Speed-Accuracy Tradeoff*	8	1	8	(17)		18956	174

**Fig. 4:**
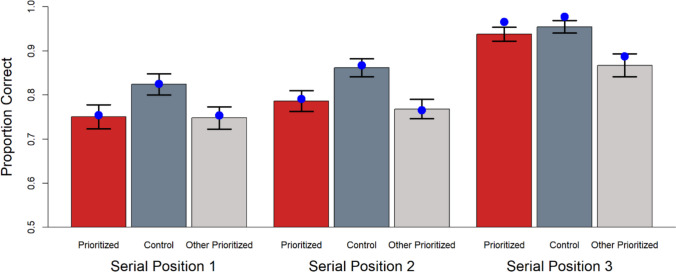
Posterior predictive plot of mean proportion correct as a function of prioritization state. Bars represent observed data. Blue dots represent data simulated from the posterior of Model 7. Error bars are 95% confidence intervals

**Fig. 5:**
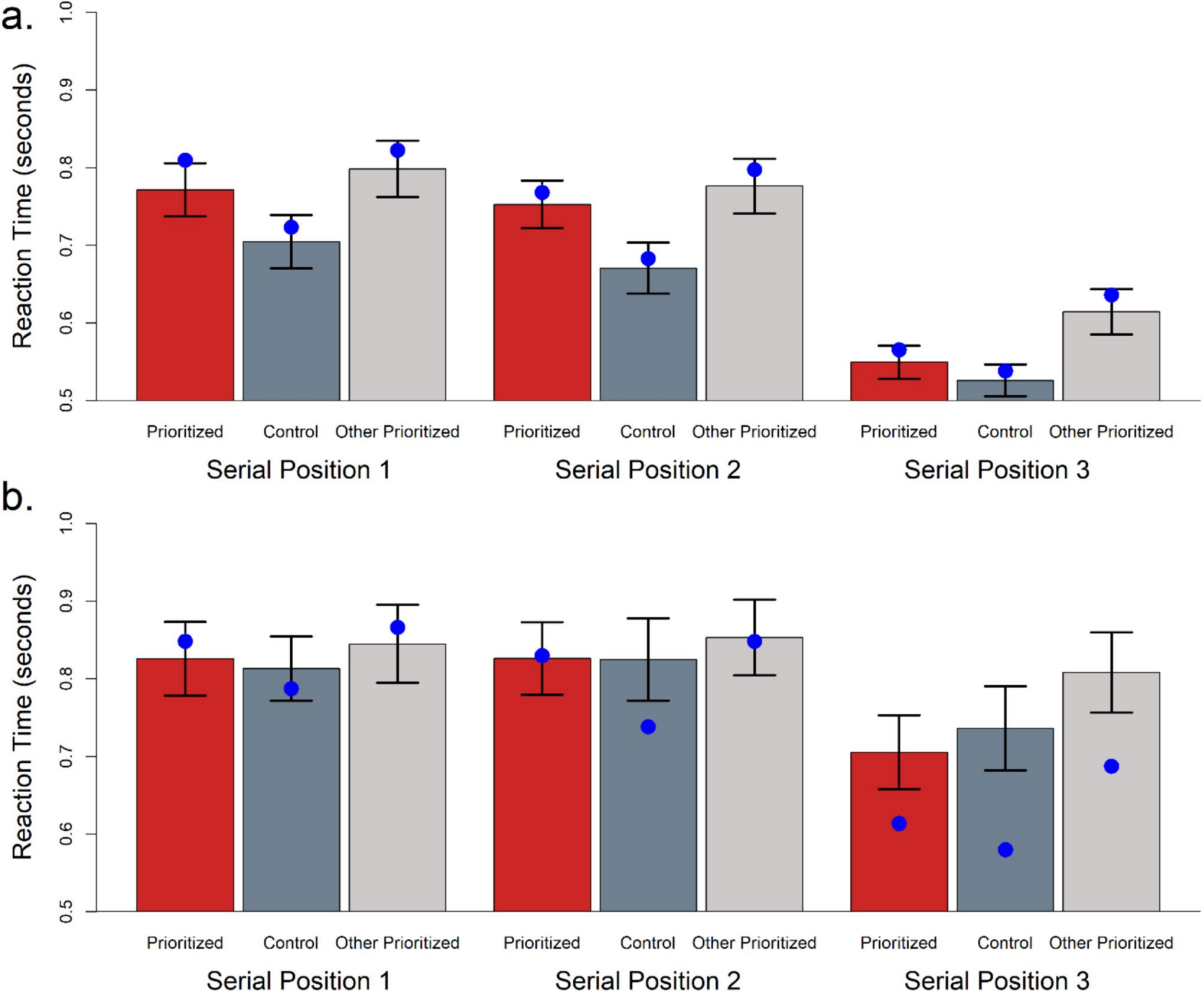
Posterior predictive plot of mean reaction time for (**a**) correct trials and (**b**) incorrect trials, as a function of prioritization state. Colored bars represent observed data. Blue dots represent data simulated from the posterior of Model 7. Error bars are 95% confidence intervals

We performed posterior predictive checks to further explore the quality of Model 7’s fit to the data. Means from the simulated data are shown as a function of serial order and prioritization state in Figs. 4 (accuracy) and 5 (RT). Full posterior predictive RT distribution plots by condition are available on the study’s OSF repository (https://osf.io/sc5g3/). The simulated data reproduce the major patterns in the observed data. The only considerable deviation from the observed data in Figs. 4 and 5 appears as faster RTs for incorrect trials when serial position 3 is tested. This is likely due to the DDM model not incorporating a guessing state. On trials when participants’ attention is off task or they miss the memory items due to blinking, they will not have sufficient information at test and must guess. This is a known source of error in brief-presentation WM tasks (Rouder et al., 2008) and results in a constant RT distribution across conditions on those trials. The impact on our observed data would be an overall flattening of the RT pattern, leading to the slower observed mean RTs for the serial position 3 responses in particular, compared to model predictions, because serial position 3 responses are particularly quick when following the drift decision process described by our model parameterization. This flattening should primarily affect the error trials as there are many fewer error trials than correct trials, making guessing trials a larger proportion of error responses.

We explored whether the inclusion of a variable boundary separation (Model 5) or more aggressive outlier trimming (removing trials from the most extreme 5% of the RT distribution) would improve the RT misfit to incorrect SP3-probed trials. Posterior predictive plots for these simulations can be found in the project’s OSF repository (https://osf.io/sc5g3/). Neither change had an appreciable effect on deviations in model fit.

**Fig. 6:**
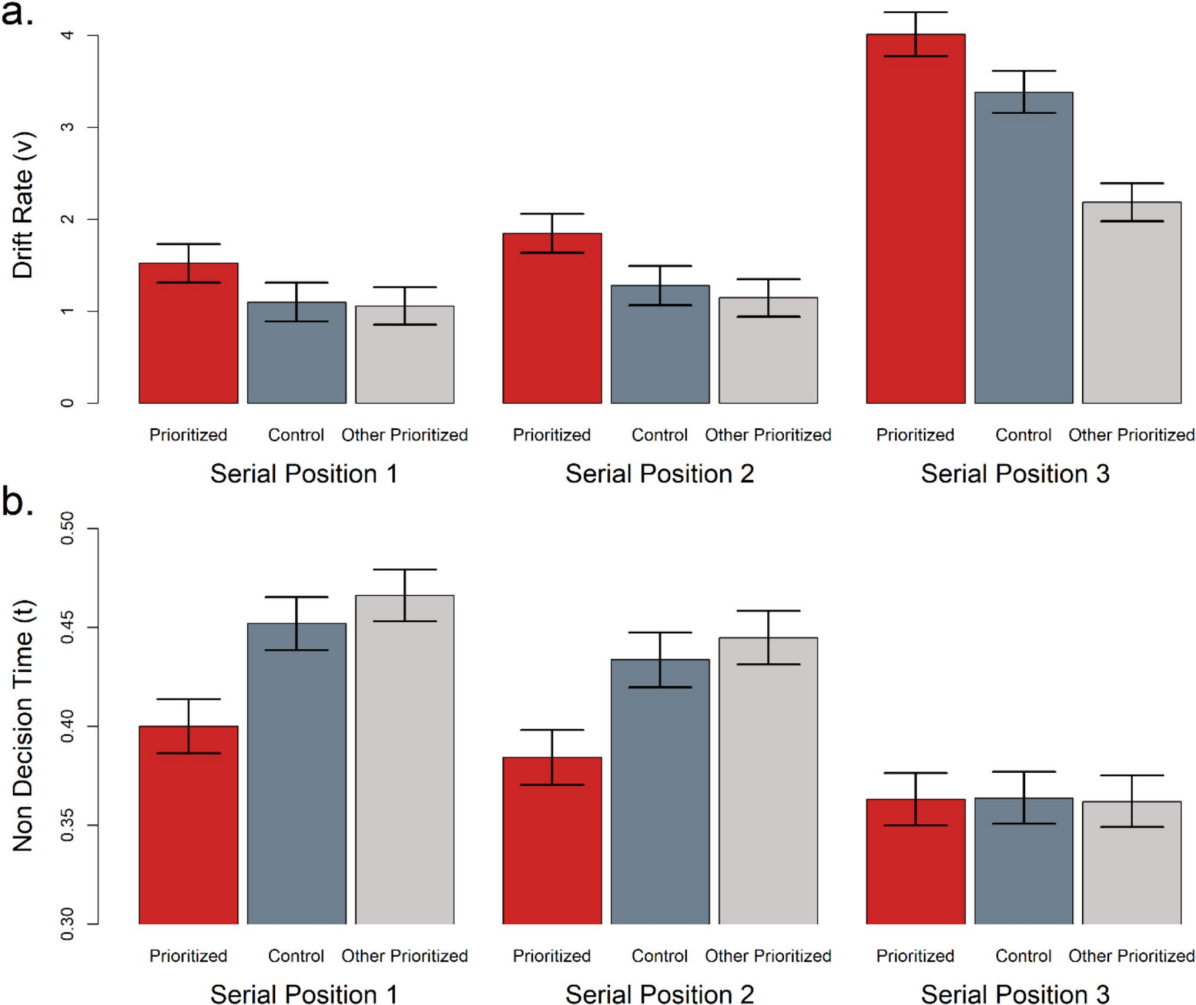
Parameter estimates as a function of prioritization and serial position from the winning model (Model 7) for (**a**) drift rate and (**b**) non-decision time. Error bars represent 95% credible intervals

Parameter estimates of drift-rate and non-decision time from Model 7 are shown in Fig. 6. When an item is prioritized, there is a clear increase in the drift rate above control levels across all serial positions (Fig. 6a), demonstrated by the prioritized and control condition means for each serial position not falling within one another’s 95% credible interval (SP1prioritized M = 1.52 [CI: 1.31, 1.73], SP1control M = 1.10 [CI: 0.89, 1.31]; SP2prioritized M = 1.85 [CI: 1.63, 2.06], SP2control M = 1.28 [CI: 1.07, 1.49]; SP3prioritized M = 4.01 [CI: 3.77, 4.25], SP3control M = 3.38 [CI: 3.15, 3.61]). When a different item is prioritized, there is a large decrease in the drift rate relative to control for the most recent item (SP3control M = 3.38 [CI: 3.15, 3.61], SP3other M = 2.18 [CI: 1.98, 2.39]), but not for the non-recent items (SP1control M = 1.10 [CI: 0.89, 1.31], SP1other M = 1.05 [CI: 0.85, 1.26]; SP2control M = 1.28 [CI: 1.07, 1.49], SP2other M = 1.15 [CI: 0.94, 1.35]).

The pattern of parameter estimates for non-decision time is dramatically different (Fig. 6b). Non-recent serial positions show faster non-decision time relative to control when prioritized (SP1prioritized M = 0.40 [CI: 0.39, 0.41], SP1control M = 0.45 [CI: 0.44, 0.47]; SP2prioritized M = 0.38 [CI: 0.37, 0.40], SP2control M = 0.43 [CI: 0.42, 0.45]) and slower non-decision time relative to control when a different item is prioritized (SP1control M = 0.45 [CI: 0.44, 0.47], SP1other M = 0.47 [CI: 0.45, 0.48]; SP2control M = 0.43 [CI: 0.42, 0.45], SP2other M = 0.44 [CI: 0.43, 0.46]). The most recent item demonstrates an especially fast non-decision time and invariance across prioritization states (SP3prioritized M = 0.36 [CI: 0.35, 0.38], SP3control M = 0.36 [CI: 0.35, 0.38], SP3other M = 0.36 [CI: 0.35, 0.38]).

## Discussion

We used a serial WM task to compare a Resource Trade-Off model against an Attentional Boost model of prioritization within WM. Modeling supports the Resource Tradeoff explanation of prioritization effects where increased resources allocated to prioritized stimuli come at the expense of non-prioritized stimuli. This is consistent with conclusions from investigations that relied heavily on response time or accuracy (Atkinson et al., 2018; Atkinson et al., 2023; Brissenden et al., 2023; Hu et al., 2014; Sandry & Ricker, 2020; Sandry et al., 2014; Sandry et al., 2020). The pattern of drift rate estimates indicates that this resource tradeoff was observed when a non-recent list item was prioritized leading to loss of performance for the recency item. Despite this, drift rates for the recency item remained greater than for non-recent items.

We also tested whether prioritization and recency effects reflect the same mechanism (entry into the FOA) and found that when prioritization is applied to the recency item, there is an additional performance benefit beyond the recency benefit alone. Drift rates under this configuration are larger than drift rates for recency in the control condition. This suggests recency and prioritization are distinct mechanisms that each contribute to performance. The pattern of parameter estimates supports this separate-mechanisms account even if we assume that entry of recency or prioritized items into the FOA is probabilistic. If prioritization and recency are probabilistic and reflect redundant FOA entry mechanisms, we should see a reduced benefit of prioritizing a recent item relative to prioritizing a non-recent item. Figure 6a shows the opposite pattern in the drift rates. A comparable or slightly larger effect of prioritizing the recency item is evident relative to the non-recency items.

Converging evidence for distinct mechanisms of recency and prioritization comes from work by Niklaus et al. (2019), who found that the recency and retro-cue effects are separate mechanisms in a series of studies characterizing the empirical speed-accuracy tradeoff with varying response deadlines. Our work extends this finding from the retro-cue paradigm, which manipulates the probability of testing an item, to reward-based prioritization. It is important not to immediately conflate reward-based and retro-cue effects on item prioritization. Reward-based prioritization often results in smaller or less consistent effects than retro-cueing (Atkinson et al., 2018; Jeanneret et al., 2023; Zhang & Lewis-Peacock, 2023). This may simply be a matter of cueing strength or perceived reward strength being smaller in some instances of reward-based cueing as the size and context of the reward changes across studies. But it remains possible that reward-based and retro cueing may differ in some or all cognitive mechanisms.

While our main conclusions are focused on the patterns in drift rates, additional information is evident when evaluating non-decision times. Figure 6b shows a striking pattern of fast and invariant non-decision time estimates for the recency item across prioritization conditions. This performance benefit also occurs for prioritized non-recent items and may reflect enhanced premotor processing. Prioritization of *non-recent* stimuli may confer an advantage by preparing the representation for use in response selection, allowing for faster responding to the probe stimulus during test. In support of this, evidence from the retro-cue literature shows that retro-cues reduce non-decision time, which has been interpreted as retro-cues providing a head start in retrieval, preceding the decision process (Shepherdson, 2020; Shepherdson et al., 2018; Souza & Frischkorn, 2023). Joseph and Morey (2022) have also argued that motor preparation for responding is the source of dual-tasking conflict during WM performance. While it is possible that prioritization influences motor response speed itself, not motor preparation, we do not see a strong theoretical reason for this within our 2AFC response paradigm.

While we suggest that speeded non-decision times may result from enhanced motor preparation, they may also reflect enhanced perceptual processing of the probe. In our task, memory items were selected because they are difficult to rehearse and preexisting conceptual representations that encapsulate the visual details do not exist for participants in our sample (Ricker et al., 2010), forcing individuals to rely on visual representation. Activation of visual features in the probe may be facilitated when a matching item is already enhanced by recent presentation or prioritization. In the future, encoding and response effects on non-decision time could be further disentangled using electromyograph and computational modeling (Weindel et al., 2021).

Taken together, the evidence supports a process in which participants passively viewed the memory items without expending more than minimal resources on the representations, unless a prioritization cue appeared. The final list item always generated a more detailed and accessible representation than earlier list items. When participants encountered a cue to prioritize an item, the representation of that item was enhanced. If the prioritized item was a non-recent item, prioritization also impaired the representation of the recency item. This may have occurred though extended drawdown of an encoding resource (Mızrak & Oberauer, 2021; Popov & Reder, 2020), extended consolidation of the earlier prioritized item (Ricker & Hardman, 2017; Wyble et al., 2011), or some other asymmetrical resource-limited process.

### What about refreshing and rehearsal?

In the past work, we interpreted prioritization in the context of attentional refreshing (Sandry & Ricker, 2020; Sandry et al., 2014; Sandry et al., 2020). In refreshing models of memory, memories are maintained by sequentially cycling items through the FOA, refreshing or enhancing the activation of each decaying memory trace (Barrouillet et al., 2004; Camos et al., 2018; Raye et al., 2007; Vergauwe & Langerock, 2017). In this approach, prioritization would bias refreshing in favor of prioritized items at the expense of non-prioritized items. This should result in prioritized items having enhanced representations (greater drift rates) and more accessible representations (faster non-decision times), both at the expense of non-prioritized items.

The parameter estimates presented in Fig. 6 argue against a refreshing mechanism in two respects. First, prioritizing the most recent item should lead to decreased refreshing of non-recent items, but Fig. 6a shows that prioritizing the recency item does not reduce drift rates for earlier list items or reduces their drift rate by such a small amount as to be theoretically inconsequential. Second, prioritizing a non-recent item should bias the FOA away from the recency item, reducing recency drift rates and non-decision time, but Fig. 6b demonstrates that the recency item always shows enhanced non-decision time. In a refreshing-based explanation this would indicate that the recency item does not leave the FOA when another item is prioritized. These patterns cannot be reconciled with a sequential refreshing mechanism.

Alternatively, the pattern of non-decision times could be interpreted through the lens of verbal rehearsal with speeded non-decision times reflecting a bias toward rehearsing those items. The difficulty with this explanation is again the invariance of non-decision times across prioritization states for the recency item while also seeing variance in the drift rates across prioritization states for the recency item. Drift rates should change in tandem with non-decision times if rehearsal is the driving factor. For this explanation to be credible one would have to assume that the invariance in recency non-decision times reflects a floor effect in perception and responding for highly rehearsed recency items. In conflict with this assumption, verbal rehearsal loops require time to initiate. This would delay engaging with the probe item, making the idea of floor effects in recency non-decision time implausible (Naveh-Benjamin & Jonides, 1984).

Further evidence against an account of prioritization as a change in refreshing/rehearsal to counteract memory loss comes from the free time literature on WM performance. Recent work has shown that changes in WM performance with free and occupied time are inconsistent with disruption of maintenance processes to counteract forgetting (Mızrak & Oberauer, 2021; Ricker & Vergauwe, 2020, 2022). These studies indicate that attention-based processing of memory items is related to encoding or consolidation of items rather than maintenance (Oberauer, 2022, 2025).

## Future directions

The present findings also open new questions. One open physiological question is whether prioritization effects are primarily due to early or late selection, which may have implications for aging and disease (Alperin et al., 2013). We have also proposed a consolidation account of prioritization (Sandry & Ricker, 2020; Sandry et al., 2014). In that model, prioritization may help stabilize the representation in WM. Biologically, prioritizing attention may interact with memory and encourage tetanic firing. Thus, directing attention via prioritization may improve the efficiency of long-term potentiation (Bliss & Collingridge, 1993; Bliss & Lømo, 1973). Some support for this model comes from evidence that prioritization within WM also improves long-term memory for prioritized positions (Sandry et al., 2020). Prioritization would act during the early process of cellular consolidation (Dudai, 2004; Genzel & Wixted, 2017; Wixted & Cai, 2013), thereby strengthening and improving the short-term consolidation process (Cotton & Ricker, 2022; Ricker, 2015; Ricker et al., 2018). Prioritization may also protect the prioritized list position against processes that interfere with short-term consolidation, for example, mask-related interference (Ricker & Sandry, 2018). Consolidation has also been proposed as a mechanism involved in the retro-cue effect (Luo et al., 2023), and so may represent a unified account of cueing benefits in WM. Additional research is necessary to further unpack relationships between prioritization, WM mechanisms, and biological processes.

## Conclusion

In the present experiment, we applied HDDM computational modeling to evaluate two alternative models of resource distribution within WM when individuals prioritize specific items. Model comparison supports a resource trade-off model with asymmetric effects across recent and non-recent items. Prioritization and recency were also found to be separate processes that together lead to a joint performance benefit.

## Supplementary Information

Below is the link to the electronic supplementary material.

Supplementary file1 (DOCX 26 KB)

Supplementary file2 (DOCX 142 KB)

## Data Availability

All data is available on the OSF at, https://osf.io/sc5g3/. Code is available upon request.

## Footnotes

1 Ratcliff used the term diffusion decision model, but we use drift-diffusion model to maintain consistency with the naming conventions of the computational modeling framework we apply in our analysis.

## Declarations

### Preregistration

The study was not preregistered.
