# Supplementary material for: Reward-based prioritization in working memory is distinct from recency and due to a resource trade-off
Source: Psychon Bull Rev. 2025 Dec 9;33(1):8. doi: 10.3758/s13423-025-02810-6 (PMC12689727; doi:10.3758/s13423-025-02810-6)
Supplement: Supplementary file 1 — Supplementary file1 (DOCX 26 KB) [file 13423_2025_2810_MOESM1_ESM.docx]

**Online Supplement 1: Behavioral Data Analysis**

We used Bayes factors for ANOVAs (Rouder et al., 2012) as our primary inferential approach to understanding changes in behavioral measures of RT and accuracy across conditions and sessions. Bayes factors presented are the BF_10_ format, the ratio between the likelihood of the data given an effect (alternative) to the likelihood of the data given no effect (null, no difference between means). In this context, a value of 51519 in support of an effect should be interpreted as the data being 51519 times more likely under the alternative than under the null. Statistical analyses were evaluated using a 3 (Session) X 4 (Prioritization Position) X 3 (Probed Position) repeated measures Bayesian ANOVA. Bayes factor calculations reported here assume that in order for an interaction to be present, the related main effects must also be included in the model. All statistical analyses were computed using the BayesFactor package (Morey & Rouder, 2024) in R version 4.0.3.

**Response Time**

As visualized in Figure 3a (main text), the preferred model for RTs included effects of Prioritization Position (F(3, 144)=4.34, η*_p_^2^*=.08, BF_10_=1.40 x 10^71^), Probed Position (F(2, 96)=183.49, η*_p_^2^*=.79, BF_10_=1.68 x 10^301^), Session (F(2, 96)=1.75, η*_p_^2^*=.04, BF_10_=2.23), and the 2-way interaction between Prioritization Position and Probed Position (F(6, 288)=39.8, η*_p_^2^*=.45, BF_10_=1.64 x 10^73^). The interaction between Prioritization Position and Probed Position reflects speeded responses to prioritized serial positions and slowed responses to non-prioritized positions (see Figure 3A). Note that the evidence in favor of Session was ambiguous but trended in the favor of an effect being present. All other interactions favored the null, Session and Probed Position (F(4, 192)=2.84, η*_p_^2^*=.06, BF_10_=0.01), Session and Prioritized Position (F(6, 288)=0.72, η*_p_^2^*=.01, BF_10_=0.03 x 10^-2^), and the 3-way interaction (F(12, 576)=0.95, η*_p_^2^*=.02, BF_10_=0.09 x 10^-8^). The RT data pattern replicated our prior investigations with shortened RTs for prioritized positions and the final list item (Sandry & Ricker, 2020; Sandry et al., 2014; Sandry et al., 2020).

**Accuracy**

As visualized in Figure 3b (main text), the preferred model for accuracies included effects of Prioritization Position (F(3, 144)=0.66, η*_p_^2^*=.01, BF_10_=1.34 x 10^56^), Probed Position (F(2, 96)=187.8, η*_p_^2^*=.80, BF_10_=9.76 x 10^183^), Session (F(2, 96)=10.66, η*_p_^2^*=.18, BF_10_= 270,063), and the 2-way interaction between Prioritization Position and Probed Position (F(6, 288)=28.79, η*_p_^2^*=.37, BF_10_=6.52 X 10^58^). The interaction between Prioritization Position and Probed Position is complex but reflects more accurate responses to prioritized serial positions and decreased accuracy for the final serial position when a non-recent item was prioritized (see Figure 3B). All other interactions favored the null, Session and Probed Position(F(4, 192)=1.70, η*_p_^2^*=.03, BF_10_=0.01),. The interaction between Session and Prioritized Position (F(6, 288)=0.94, η*_p_^2^*=.02, BF_10_=0.08 x 10^-2^), and the 3-way interaction (F(12, 576)=0.71, η*_p_^2^*=.01, BF_10_=4.35 x 10^-9^). The accuracy data pattern replicated our prior investigations with high accuracy for prioritized positions and the final list item (Sandry & Ricker, 2020; Sandry et al., 2014; Sandry et al., 2020).

**References**

Morey, R., & Rouder, J. (2024). Bayes Factor: Computation of Bayes factors for simple designs (Version 0.9.12-4.7)[Computer program]. ]. Retrieved from <http://CRAN.R-project.org/package=BayesFactor>. In.

Rouder, J. N., Morey, R. D., Speckman, P. L., & Province, J. M. (2012). Default Bayes factors for ANOVA designs. *Journal of Mathematical Psychology*, *56*(5), 356-374.

Sandry, J., & Ricker, T. J. (2020). Prioritization within visual working memory reflects a flexible focus of attention. *Attention, Perception, & Psychophysics*. <https://doi.org/10.3758/s13414-020-02049-4>

Sandry, J., Schwark, J. D., & MacDonald, J. (2014). Flexibility within working memory and the focus of attention for sequential verbal information does not depend on active maintenance. *Memory & Cognition*, *42*, 1130-1142. <https://doi.org/10.3758/s13421-014-0422-1>

Sandry, J., Zuppichini, M. D., & Ricker, T. J. (2020). Attentional flexibility and prioritization improves long-term memory. *Acta Psychologica*, *208*, 103104. <https://doi.org/https://doi.org/10.1016/j.actpsy.2020.103104>
