# Supplementary material for: Reward-based prioritization in working memory is distinct from recency and due to a resource trade-off
Source: Psychon Bull Rev. 2025 Dec 9;33(1):8. doi: 10.3758/s13423-025-02810-6 (PMC12689727; doi:10.3758/s13423-025-02810-6)
Supplement: Supplementary file 2 — Supplementary file2 (DOCX 142 KB) [file 13423_2025_2810_MOESM2_ESM.docx]

**Online Supplement 2: Post Task Questionnaires**

Reward can be used to induce prioritization, and one outstanding question is whether individuals vary in susceptibility to reward-related prioritization. Reward influences selective attention (Anderson, 2013; Engelmann et al., 2009; Engelmann & Pessoa, 2014; Krebs et al., 2011; Small et al., 2005), stimulus prioritization during encoding (Miendlarzewska et al., 2016), and WM (Gilbert & Fiez, 2004; Jimura et al., 2010; Krawczyk et al., 2007; Morey et al., 2011; Taylor et al., 2004). Trait-level differences in reward sensitivity across individuals influence executive function (Capa & Bouquet, 2018) and may directly influence prioritization within the FOA. Another potential person-specific factor is the tendency to engage in mind-wandering, which influences cognition (Mooneyham & Schooler, 2013) and WM performance (Cotton et al., 2023; Kane et al., 2007; Krimsky et al., 2017). In the current study, we include measures of reward sensitivity and mind-wandering to determine whether individual tendencies modulate the ability to prioritize memoranda.

Our design required participants to return to the lab for a total of 3 visits on different days, each one week apart. It is possible that performance will vary across sessions because of individual differences across different visits. For example, if participants are more tired during one session in contrast to other sessions, they may rely on using a more passive WM maintenance strategy in contrast to an active WM maintenance strategy. While there is not a strong theoretical rationale to assume differential strategy use across sessions, we included a measure of self-reported strategy use used in our prior prioritization research (adapted from Morrison et al., 2016; Sandry & Ricker, 2020). Our rationale was to investigate differences in patterns of strategy use across sessions in more detail, as this may provide insight into how strategy may impact prioritization. To foreshadow, there were no interactive effects of Session, thus, we only present descriptive strategy data.

We used Bayes factors for ANOVAs (Rouder et al., 2012) and t-tests (Rouder et al., 2009) to examine changes in participant responses. Bayes factors presented are the BF_10_ format, the ratio between the likelihood of the data given an effect (alternative) to the likelihood of the data given no effect (null, no difference between means). In this context, a value of 51519 in support of an effect should be interpreted as the data being 51519 times more likely under the alternative than under the null. All statistical analyses were computed using the BayesFactor package (Morey & Rouder, 2024) in R version 4.0.3.

**Self-Report Questions**

Self-reported motivation responses aggregated across participants for each session are visualized in Supplemental Figure 1. There was moderate evidence supporting a decrease in motivation to score points across sessions, F(2,135)=3.27, η*_p_^2^*=.05, BF=7.3. This was primarily driven by a small difference between the first and third sessions, t(45)=3.20, BF=4.8.

There was strong evidence supporting an increase in tiredness across sessions, F(2,126)=6.27, η*_p_^2^*=.09, BF=1362. This was primarily driven by an increase in tiredness/fatigue between the first and second sessions, t(42)=4.35, BF=5.4, but no difference across the second and third sessions, BF=.26.

There was ambiguous evidence trending toward a change in overall motivation across sessions, F(2,120)=2.46, η*_p_^2^*=.04, BF=2.8. This weak effect was driven by a small decrease between the first and third session, t(40)=2.72, BF=1.74, the evidence for a difference between the first and second sessions was ambiguous, BF=.66.

**Supplemental Figure 1.**

**
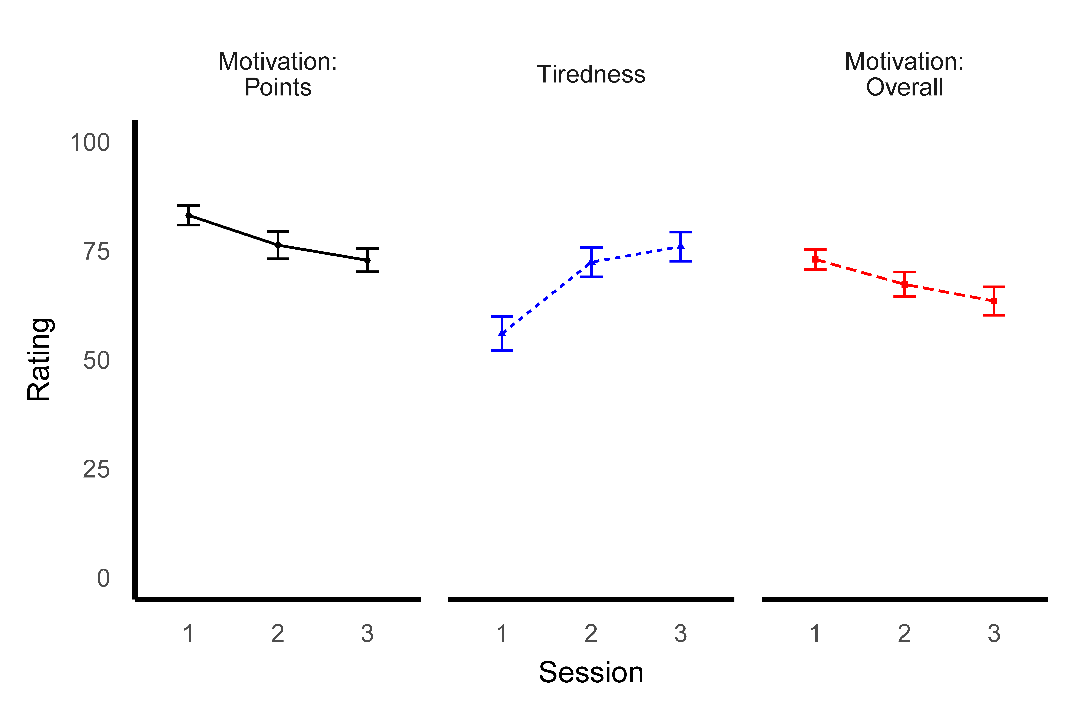
***Mean self-report ratings (0 - Not at All to 100 - Extremely) for “How motivated were you to score a high number of points?”, “How tired were you while performing the experiment?”, and “Overall, how motivated were you throughout the experiment?”*

**Strategy Use**

Strategy use across sessions is visualized in Supplemental Figure 2. Glossary definitions of the different strategies are available in Sandry and Ricker (2020). Only a few participants reported using a rehearsal strategy (Session 1 = 8%, Session 2= 2%, Session 3=6%). Association and Concentration strategies were reported as the most frequently adopted within each session. This corroborates our prior research, where we found only 5% of the sample reported adopting a rehearsal strategy under the same experimental conditions and stimulus materials, verifying that verbal recoding through use of a rehearsal strategy is an unlikely alternative maintenance explanation for the prioritization effect.

**Supplemental Figure 2.**

*Proportion of participants endorsing different working memory maintenance strategies across Sessions 1, 2 & 3. Two bottom facets represent replotted responses from Experiment 3 of Sandry & Ricker (2020) broken down by between-participant conditions.*





**Correlations**

We computed Pearson correlations between drift rate parameter estimates derived from the best fitting model, and self-reported reward responsiveness (BIS/BAS) and mind-wandering. While four subscales are included on the BIS/BAS, we only evaluated correlations with the reward responsiveness subscale given our hypotheses are specific to reward (Bonferroni adjustment [α=.05/9=.006]).

There was no large or systematic pattern of correlation between reward responsiveness or self-reported mind-wandering and the model 7 parameter estimates, but a few small correlations were significant (see supplemental tables 1 & 2). Given the large number of correlations estimated in this analysis, the small size of the correlations, and the lack of a clear pattern we do not interpret these further.

**Supplemental Table 1**

*Correlations with confidence intervals between Model 7 DDM (v) parameter estimates & self-reported mind wandering (MWQ) and reward responsiveness (BIS/BAS).*

| Variable | 1 | 2 | 3 | 4 | 5 | 6 | 7 | 8 | 9 | 10 |  |  |
| --- | --- | --- | --- | --- | --- | --- | --- | --- | --- | --- | --- | --- |
|  |  |  |  |  |  |  |  |  |  |  |  |  |
| 1. MWQ |  |  |  |  |  |  |  |  |  |  |  |  |
|  |  |  |  |  |  |  |  |  |  |  |  |  |
| 2. Reward Responsiveness | .01 |  |  |  |  |  |  |  |  |  |  |  |
|  | [-.16, .17] |  |  |  |  |  |  |  |  |  |  |  |
|  |  |  |  |  |  |  |  |  |  |  |  |  |
| 3. sp1_cntrl | .17* | .02 |  |  |  |  |  |  |  |  |  |  |
|  | [.01, .32] | [-.14, .18] |  |  |  |  |  |  |  |  |  |  |
|  |  |  |  |  |  |  |  |  |  |  |  |  |
| 4. sp1_in | .04 | -.03 | -.02 |  |  |  |  |  |  |  |  |  |
|  | [-.13, .20] | [-.19, .13] | [-.18, .14] |  |  |  |  |  |  |  |  |  |
|  |  |  |  |  |  |  |  |  |  |  |  |  |
| 5. sp1_out | .07 | .01 | .74** | -.08 |  |  |  |  |  |  |  |  |
|  | [-.09, .23] | [-.15, .18] | [.66, .81] | [-.23, .09] |  |  |  |  |  |  |  |  |
|  |  |  |  |  |  |  |  |  |  |  |  |  |
| 6. sp2_cntrl | .14 | -.00 | .55** | .10 | .60** |  |  |  |  |  |  |  |
|  | [-.02, .29] | [-.16, .16] | [.43, .66] | [-.06, .26] | [.49, .70] |  |  |  |  |  |  |  |
|  |  |  |  |  |  |  |  |  |  |  |  |  |
| 7. sp2_in | .03 | -.03 | -.09 | .73** | -.26** | .06 |  |  |  |  |  |  |
|  | [-.13, .19] | [-.19, .13] | [-.25, .07] | [.65, .80] | [-.41, -.11] | [-.11, .22] |  |  |  |  |  |  |
|  |  |  |  |  |  |  |  |  |  |  |  |  |
| 8. sp2_out | .18* | .01 | .67** | -.19* | .79** | .72** | -.21* |  |  |  |  |  |
|  | [.02, .33] | [-.16, .17] | [.57, .75] | [-.34, -.03] | [.72, .84] | [.64, .79] | [-.36, -.05] |  |  |  |  |  |
|  |  |  |  |  |  |  |  |  |  |  |  |  |
| 9. sp3_cntrl | .08 | -.01 | .36** | .13 | .38** | .34** | .16 | .36** |  |  |  |  |
|  | [-.09, .24] | [-.17, .15] | [.21, .49] | [-.04, .28] | [.23, .51] | [.19, .48] | [-.00, .31] | [.21, .49] |  |  |  |  |
|  |  |  |  |  |  |  |  |  |  |  |  |  |
| 10. sp3_in | .06 | -.02 | .02 | .61** | -.03 | .24** | .57** | .01 | .56** |  |  |  |
|  | [-.10, .22] | [-.18, .14] | [-.14, .18] | [.50, .70] | [-.19, .14] | [.09, .39] | [.45, .67] | [-.16, .17] | [.43, .66] |  |  |  |
|  |  |  |  |  |  |  |  |  |  |  |  |  |
| 11. sp3_out | .18* | .01 | .34** | -.38** | .58** | .44** | -.52** | .69** | .47** | -.01 |  |  |
|  | [.02, .33] | [-.15, .17] | [.19, .48] | [-.51, -.23] | [.46, .68] | [.30, .56] | [-.62, -.39] | [.60, .77] | [.34, .59] | [-.17, .15] |  |  |
|  |  |  |  |  |  |  |  |  |  |  |  |  |

*Note.* Values in square brackets indicate the 95% confidence interval for each correlation. * indicates *p* < .05. ** indicates *p* < .01. No correlations between individual differences in MWQ and Reward Responsiveness and DDM parameters were significant after Bonferroni adjustment [.05/9=.006].

**Supplemental Table 2**

*Correlations with confidence intervals between Model 7 DDM (t) parameter estimates & self-reported mind wandering (MWQ) and reward responsiveness (BIS/BAS).*

| Variable | 1 | 2 | 3 | 4 | 5 | 6 | 7 | 8 | 9 | 10 |  |  |
| --- | --- | --- | --- | --- | --- | --- | --- | --- | --- | --- | --- | --- |
|  |  |  |  |  |  |  |  |  |  |  |  |  |
| 1. MWQ |  |  |  |  |  |  |  |  |  |  |  |  |
|  |  |  |  |  |  |  |  |  |  |  |  |  |
| 2. Reward Responsiveness | .01 |  |  |  |  |  |  |  |  |  |  |  |
|  | [-.16, .17] |  |  |  |  |  |  |  |  |  |  |  |
|  |  |  |  |  |  |  |  |  |  |  |  |  |
| 3. sp1_cntrl | .01 | .01 |  |  |  |  |  |  |  |  |  |  |
|  | [-.15, .17] | [-.16, .17] |  |  |  |  |  |  |  |  |  |  |
|  |  |  |  |  |  |  |  |  |  |  |  |  |
| 4. sp1_in | .25** | .02 | .40** |  |  |  |  |  |  |  |  |  |
|  | [.09, .39] | [-.14, .18] | [.26, .53] |  |  |  |  |  |  |  |  |  |
|  |  |  |  |  |  |  |  |  |  |  |  |  |
| 5. sp1_out | .10 | -.01 | .82** | .25** |  |  |  |  |  |  |  |  |
|  | [-.06, .26] | [-.17, .15] | [.76, .87] | [.10, .40] |  |  |  |  |  |  |  |  |
|  |  |  |  |  |  |  |  |  |  |  |  |  |
| 6. sp2_cntrl | .03 | .02 | .73** | .55** | .64** |  |  |  |  |  |  |  |
|  | [-.13, .19] | [-.14, .18] | [.64, .79] | [.43, .66] | [.54, .73] |  |  |  |  |  |  |  |
|  |  |  |  |  |  |  |  |  |  |  |  |  |
| 7. sp2_in | .25** | .03 | .31** | .85** | .13 | .59** |  |  |  |  |  |  |
|  | [.09, .40] | [-.13, .19] | [.16, .45] | [.80, .89] | [-.03, .28] | [.47, .68] |  |  |  |  |  |  |
|  |  |  |  |  |  |  |  |  |  |  |  |  |
| 8. sp2_out | .10 | .00 | .74** | .41** | .76** | .79** | .42** |  |  |  |  |  |
|  | [-.06, .26] | [-.16, .16] | [.65, .80] | [.27, .54] | [.68, .82] | [.72, .84] | [.28, .55] |  |  |  |  |  |
|  |  |  |  |  |  |  |  |  |  |  |  |  |
| 9. sp3_cntrl | -.06 | -.01 | .27** | .51** | .17* | .56** | .67** | .40** |  |  |  |  |
|  | [-.22, .10] | [-.17, .15] | [.11, .41] | [.38, .62] | [.01, .33] | [.44, .66] | [.57, .75] | [.25, .53] |  |  |  |  |
|  |  |  |  |  |  |  |  |  |  |  |  |  |
| 10. sp3_in | -.02 | .00 | .23** | .51** | .17* | .54** | .67** | .42** | .91** |  |  |  |
|  | [-.18, .15] | [-.16, .16] | [.07, .38] | [.38, .62] | [.01, .33] | [.42, .65] | [.58, .75] | [.28, .55] | [.88, .93] |  |  |  |
|  |  |  |  |  |  |  |  |  |  |  |  |  |
| 11. sp3_out | -.02 | -.02 | .22** | .32** | .21* | .45** | .48** | .42** | .80** | .87** |  |  |
|  | [-.18, .14] | [-.18, .14] | [.06, .37] | [.17, .46] | [.05, .36] | [.31, .57] | [.35, .60] | [.28, .55] | [.74, .85] | [.82, .90] |  |  |
|  |  |  |  |  |  |  |  |  |  |  |  |  |

*Note.* Values in square brackets indicate the 95% confidence interval for each correlation. * indicates *p* < .05. ** indicates *p* < .01. No correlations between individual differences in MWQ and Reward Responsiveness and DDM parameters were significant after Bonferroni adjustment [.05/9=.006].

**References**

Anderson, B. A. (2013). A value-driven mechanism of attentional selection. *Journal of vision*, *13*(3), 7-7.

Capa, R. L., & Bouquet, C. A. (2018). Individual Differences in Reward Sensitivity Modulate the Distinctive Effects of Conscious and Unconscious Rewards on Executive Performance [Original Research]. *Frontiers in psychology*, *9*. <https://doi.org/10.3389/fpsyg.2018.00148>

Cotton, K., Sandry, J., & Ricker, T. J. (2023). Secondary task engagement drives the McCabe effect in long-term memory. *Memory & cognition*, 1-13.

Engelmann, J. B., Damaraju, E., Padmala, S., & Pessoa, L. (2009). Combined effects of attention and motivation on visual task performance: transient and sustained motivational effects. *Frontiers in Human Neuroscience*, *3*, 4.

Engelmann, J. B., & Pessoa, L. (2014). Motivation sharpens exogenous spatial attention. *Motivation Science*, *1*(S), 64-72.

Gilbert, A. M., & Fiez, J. A. (2004). Integrating rewards and cognition in the frontal cortex. *Cognitive, Affective, & Behavioral Neuroscience*, *4*(4), 540-552.

Jimura, K., Locke, H. S., & Braver, T. S. (2010). Prefrontal cortex mediation of cognitive enhancement in rewarding motivational contexts. *Proceedings of the National Academy of Sciences*, *107*(19), 8871-8876.

Kane, M. J., Brown, L. H., McVay, J. C., Silvia, P. J., Myin-Germeys, I., & Kwapil, T. R. (2007). For whom the mind wanders, and when: an experience-sampling study of working memory and executive control in daily life. *Psychol Sci*, *18*(7), 614-621. <https://doi.org/10.1111/j.1467-9280.2007.01948.x>

Krawczyk, D. C., Gazzaley, A., & D'Esposito, M. (2007). Reward modulation of prefrontal and visual association cortex during an incentive working memory task. *Brain research*, *1141*, 168-177.

Krebs, R. M., Boehler, C. N., Roberts, K. C., Song, A. W., & Woldorff, M. G. (2011). The involvement of the dopaminergic midbrain and cortico-striatal-thalamic circuits in the integration of reward prospect and attentional task demands. *Cerebral cortex*, *22*(3), 607-615.

Krimsky, M., Forster, D. E., Llabre, M. M., & Jha, A. P. (2017). The influence of time on task on mind wandering and visual working memory. *Cognition*, *169*, 84-90. <https://doi.org/10.1016/j.cognition.2017.08.006>

Miendlarzewska, E. A., Bavelier, D., & Schwartz, S. (2016). Influence of reward motivation on human declarative memory. *Neuroscience & Biobehavioral Reviews*, *61*, 156-176.

Mooneyham, B. W., & Schooler, J. W. (2013). The costs and benefits of mind-wandering: a review. *Canadian Journal of Experimental Psychology/Revue canadienne de psychologie expérimentale*, *67*(1), 11.

Morey, C. C., Cowan, N., Morey, R. D., & Rouder, J. N. (2011). Flexible attention allocation to visual and auditory working memory tasks: Manipulating reward induces a trade-off. *Attention, Perception, & Psychophysics*, *73*(2), 458-472.

Morey, R., & Rouder, J. (2024). Bayes Factor: Computation of Bayes factors for simple designs (Version 0.9.12-4.7)[Computer program]. ]. Retrieved from <http://CRAN.R-project.org/package=BayesFactor>. In.

Morrison, A. B., Rosenbaum, G. M., Fair, D., & Chein, J. M. (2016). Variation in strategy use across measures of verbal working memory. *Memory & cognition*, *44*(6), 922-936.

Rouder, J. N., Morey, R. D., Speckman, P. L., & Province, J. M. (2012). Default Bayes factors for ANOVA designs. *Journal of Mathematical Psychology*, *56*(5), 356-374.

Rouder, J. N., Speckman, P. L., Sun, D., Morey, R. D., & Iverson, G. (2009). Bayesian t tests for accepting and rejecting the null hypothesis. *Psychonomic bulletin & review*, *16*(2), 225-237.

Sandry, J., & Ricker, T. J. (2020). Prioritization within visual working memory reflects a flexible focus of attention. *Attention, Perception, & Psychophysics*. <https://doi.org/10.3758/s13414-020-02049-4>

Small, D. M., Gitelman, D., Simmons, K., Bloise, S. M., Parrish, T., & Mesulam, M.-M. (2005). Monetary incentives enhance processing in brain regions mediating top-down control of attention. *Cerebral cortex*, *15*(12), 1855-1865.

Taylor, S. F., Welsh, R. C., Wager, T. D., Phan, K. L., Fitzgerald, K. D., & Gehring, W. J. (2004). A functional neuroimaging study of motivation and executive function. *Neuroimage*, *21*(3), 1045-1054.
